# Mitochondrial DNA sequence of the hybrid of *Pelteobagrus vachelli* (♀) × *Pelteobagrus fulvidraco* (♂)

**DOI:** 10.1080/23802359.2019.1674741

**Published:** 2019-10-09

**Authors:** Tingshuang Pan, Jun Ling, Guoqing Duan, He Jiang

**Affiliations:** a Fisheries Institute, Anhui Academy of Agricultural Sciences, Hefei, Anhui, China;; b Anhui Province Key Laboratory of Aquaculture & Stock Enhancement, Hefei, Anhui, China

**Keywords:** *Pelteobagrus vachelli*, hybrid, *Pelteobagrus fulvidraco*

## Abstract

The complete mitochondrial genome sequence of a hybrid, which was produced by sexual hybridization of *Pelteobagrus vachelli* (♀) × *Pelteobagrus fulvidraco* (♂), was obtained. The complete mitochondrial genome of the hybrid is 16,532 bp, containing 37 genes. 129 Site differences were found in overall length compared with *P. vachelli*.

*Pelteobagrus vachelli* and *Pelteobagrus fulvidraco* both belong to Bagridae. In this study, a hybrid was produced by female *P. vachelli* and male *P. fulvidraco*. Moreover, the complete mitochondrial genome of the hybrid was sequenced and compared with *P. vachelli* and constructed a phylogenetic tree by 13 protein-coding genes.

The hybrid individuals were collected from Fisheries Institute, Anhui Academy of Agricultural Sciences, Hefei, China (31°53′33″N, 117°15′9″E). Voucher specimen (HB20190530-6) was deposited in Fish Specimen Library at Fisheries Institute Anhui Academy of Agriculture Sciences, Anhui, China. We amplified mitochondrial DNA using PCR method as reported (Pan et al. 2019). The complete mitochondrial genome of the hybrid is 16,532 bp (MN365880), containing a total of 37 genes. The 13 protein-coding genes in the mitochondrial genome of the hybrid also share features in start and stop codons with those in other fishes in Bagridae (Liang et al. 2013; Liang et al. 2016). Compared with the mitochondrial sequence of *P. vachelli* (MK134689), 129 Site differences were found between their mitochondrial genome. About 8 site differences were located in rRNAs, 1 in tRNAs, 9 in control region, and 111 in protein coding genes. Furthermore, 111varial bases of the protein coding regions resulted in 18 variable amino acids, which were located in *ND1*, *COX2*, *ATP6*, *COX3*, *ND4*, and *ND5*.

Phylogenetic analysis was performed using 13 protein-coding genes by neighbor-joining (NJ). The results showed that the hybrid of *P. vachelli* (♀) × *P. fulvidraco* (♂) were genetically closer with *P. vachelli* than *P. fulvidraco* (Figure 1).

**Figure 1.**
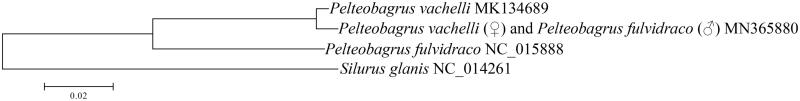
Neighbor-joining phylogenetic tree inferred from amino acid sequence dataset of 13 protein-coding genes. The tree shows the topology based on concatenated data of 13 mitochondrial encoded protein sequences. Reconstruction was performed by MEGA X (64-bit).
